# Indeterminate cell histiocytosis with ETV3::NCOA2 fusion detected by optical genome mapping^؆^

**DOI:** 10.1016/j.abd.2026.501342

**Published:** 2026-04-28

**Authors:** Miguel Mansilla-Polo, Rafael Andreu-Lapiedra, Irene Luna-del Valle, Esperanza Such-Taboada, Montserrat Évole-Buselli

**Affiliations:** a Department of Dermatology, Hospital Universitario y Politécnico La Fe, Valencia, Spain; b Instituto de Investigación Sanitaria La Fe, Valencia, Spain; c Department of Hematology, Hospital Universitario y Politécnico La Fe, Valencia, Spain; d Centro de Investigación Biomédica en Red, Instituto de Salud Carlos III, Madrid, Spain

Dear Editor,

Following observation of a generalized eruption in a young patient with a unique histopathology, the term Indeterminate Cell Histiocytosis (ICH) was described and coined by Wood GS et al. in 1985.^1^ It is a rare cutaneous proliferative disorder characterized by a proliferation of CD1a + and CD207/langerin- mononuclear phagocytic cells, lacking Birbeck granules on electron microscopy. It is currently included in Group L of the 2016 revised classification of histiocytosis, catalogued as “indeterminate dendritic cell histiocytosis” and as “indeterminate dendritic cell tumor” in the 2022 WHO classification of dendritic cell neoplasms.^2^ This classification has been reaffirmed in the 5th edition (2024) of the WHO Classification of Haematolymphoid Tumours.^3^ Recent advances have shown that a subset of patients carries the ETV3::NCOA2 fusion gene, generally detected by Fluorescence In Situ Hybridization (FISH) or Next-Generation Sequencing (NGS).^4^, ^5^, ^6^ In this paper, we report a case of a patient with florid ICH in which the ETV3::NCOA2 fusion was identified for the first time using Optical Genome Mapping (OGM), achieving a favorable outcome after treatment with cladribine.

A 17-year-old male presented with rapidly progressing cutaneous lesions over the last 6-months. Physical examination revealed numerous flesh-colored papules with a generalized, cobblestone-like distribution, respecting the acrofacial regions (Fig. 1A‒C). There were no lymphadenopathies, mucosal involvement, or systemic symptoms. Laboratory tests, including blood count, autoimmunity (ANA, myositis, and sclerosis blots), blood smear, and serologies, were normal. Histopathology (Fig. 1D) showed a dermal infiltrate that dissected the collagen fibers, consisting mainly of dense histiocytoid cells, with few lymphocytes and no eosinophils. Immunohistochemistry, the tumor cells were positive for CD1a (Fig. 2A) and S100, but negative for CD207/langerin (Fig. 2B). His clinical presentation and histopathological findings supported the diagnosis of ICH.

**Fig. 1:**
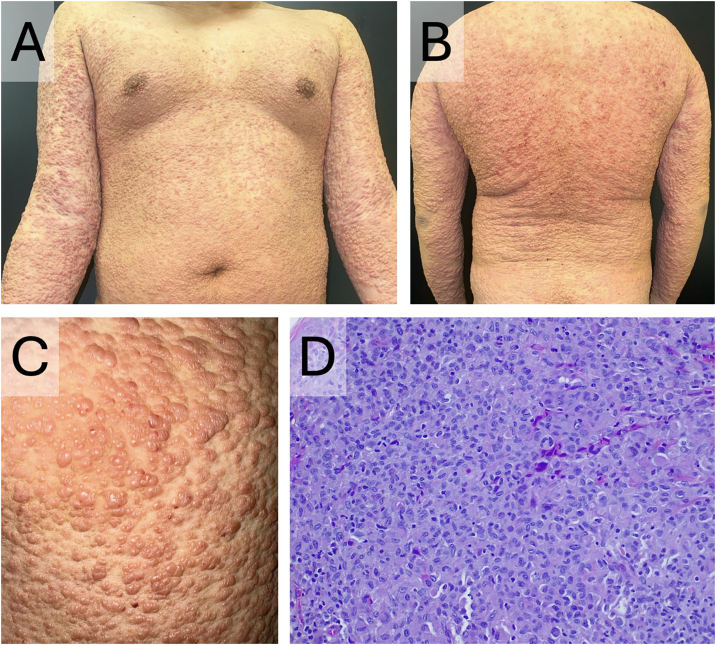
Clinical presentation of the lesions and histopathological examination of one of the lesions. (A‒B) Generalized flesh-colored papules with a 'cobblestone' appearance of the skin. (C) Close-up view of the lesions. (D) Dermal infiltrate, predominantly histiocytic, with few lymphocytes, plasma cells and no eosinophils (Hematoxylin & eosin, ×200).

**Fig. 2:**
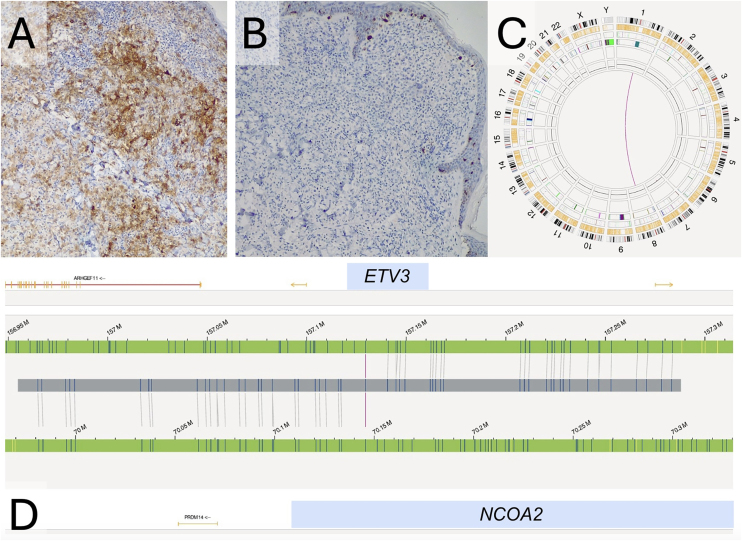
Immunohistochemistry of one of the lesions and optical genomic mapping of the tumor sample. (A) Diffuse positivity for CD1a (CD1a, 10×). (B) Negativity for CD207/langerin (CD207/langerin, 10×). (C and D) Graphical representation of the Optical Genomic Mapping with the interchromosomal translocation t(1;8) (q23.1;q13.3).

As part of the extension study, a positron emission tomography-computed tomography scan and bone marrow biopsy were performed, both of which were normal. A NGS study using the Ampliseq™ Comprehensive Cancer Panel was performed on DNA from the surgical specimen, covering more than 400 genes, including *BRAF, KRAS, NRAS, MAP2K1, NTRK1-3*, and was negative except for the presence of the variants c.1288C > T p.(Pro430Ser) in the MLLT10 gene, with a Variant Allele Frequency (VAF) of 49.7% and the variant c.49997 G > A p.(Gly1666Asp) in the *RNF213* gene, with a VAF of 43.4%, both of them of unknown clinical relevance.

Subsequently, an OGM analysis was performed on a papular specimen, which had been previously snap-frozen in liquid nitrogen at −196 °C for 3-minutes and subsequently stored at −80 °C. Data analysis was conducted using Bionano Access® Software (Bionano Genomics), applying the “Rare Variant Analysis” pipeline with the GRCh38 genome as reference. This analysis (Fig. 2C‒D) identified an interchromosomal translocation t(1;8)(q23.1;q13.3) with a VAF of 35%, leading to the formation of the ETV3::NCOA2 fusion gene.

The patient was started on prednisone 30 mg/d and methotrexate 15 mg/week. However, due to progression of the lesions after two months, treatment with cladribine 5 mg/m^2^/day (days 0‒5) was started for 4-months. He experienced a complete response of the lesions with no adverse effects or relapse at 8-month follow-up.

In recent years, advances in genetics and molecular biology techniques have led to the identification of two subtypes of ICH. The first is characterized by a predominance of mutations in the *BRAF, KRAS*, and *MAP2K1* pathways and is often associated with multisystemic disease and other hematological disorders (especially chronic myelomonocytic leukemia). The second, defined by the presence of the ETV3::NCOA2 fusion gene (detected by FISH, NGS or, in our case, by OGM), generally has a more indolent course and a favorable prognosis.^2^, ^5^, ^6^

This case highlights the utility of OGM for detecting cryptic structural variants that may go unnoticed by conventional techniques, providing valuable diagnostic and prognostic information.^7^

Numerous treatments have been reported, with surgical excision generally being the first choice for localized cutaneous forms, and methotrexate, phototherapy or chemotherapeutic agents in monotherapy (cyclophosphamide, busulfan, cladribine, vinblastine) for extensive cutaneous forms. Polychemotherapy regimens and/or hematopoietic cell transplantation are typically preferred for progressive cutaneous or multisystem involvement.^4^, ^5^

## ORCID ID

Rafael Andreu-Lapiedra: 0000-0001-8506-2187

Irene Luna-del Valle: 0000-0001-5081-8589

Esperanza Such-Taboada: 0000-0001-6892-403X

Montserrat Évole-Buselli: 0000-0002-9875-4235

## Financial support

No specific funding was received from any bodies in the public, commercial or not-for-profit sectors to carry out the work described in this article.

Authors' contributions

Miguel Mansilla-Polo: Directed the diagnosis and management of the patients and the initial writing of the manuscript.

Rafael Andreu-La Piedra: Directed the diagnosis and management of the patients and the initial writing of the manuscript.

Montserrat Évole-Buselli: Directed the diagnosis and management of the patients and the initial writing of the manuscript.

Esperanza Such-Taboada: Performed the genetics study, the final writing of the manuscript and supervised the work.

Irene Luna-del Valle: Performed the genetics study, the final writing of the manuscript and supervised the work.

## Research data availability

Does not apply.

## Conflicts of interest

None declared.
